# Teaching Health Informatics in a Skills-First Era: A Curriculum Mapping Approach Aligned With Workforce Demand

**DOI:** 10.2196/99637

**Published:** 2026-07-30

**Authors:** Jasmine Agnew

**Affiliations:** 1 Department of Healthcare Administration and Informatics, School of Public Health, Samford University, 800 Lakeshore Blvd, Birmingham, AL, 35229, United States, 1 2057264570

**Keywords:** health informatics, workforce alignment, competency-based education, skills, education, workforce, curriculum mapping

## Abstract

Academic programs in health informatics have been urged to align curricula with evolving workforce needs to better prepare graduates for practice. Such alignment supports workforce readiness, addresses talent shortages, and reduces onboarding burdens for employers. Although employers increasingly seek graduates who can demonstrate applied skills and competencies beyond textbook knowledge, academic programs continue to face challenges in translating workforce demand into curricular design. This Viewpoint is directed at health informatics program faculty and administrators and presents the author’s perspective on a skills-first curriculum mapping approach aligned with current industry needs. The author argues that connecting program-level outcomes, course objectives, and learning activities to industry-recognized competencies strengthens workforce readiness while maintaining academic rigor. Drawing on the literature on competency-based education, workforce development, and health informatics training, this paper argues for intentional curriculum design that integrates transferable skills, stackable credentials, and stakeholder engagement. The key messages are as follows: (1) workforce demand should anchor curriculum design in health informatics programs, (2) curriculum mapping is a practical mechanism for operationalizing skills-first alignment, and (3) credential integration and continuous congruency evaluation support long-term program sustainability. This approach positions health informatics education to remain responsive, scalable, and aligned with the rapidly evolving professional landscape.

## Introduction: Health Informatics in a Skills-First Workforce

In 2025, the Organisation for Economic Co-operation and Development (OECD) released a skills study exploring hiring and workforce development practices that prioritize a candidate’s demonstrated skills and competencies over their acquisition of degrees or job titles [1]. This shift aligns with a fast-changing workforce in which agility is integral to ensuring sustainability. With consistent clinical and technical changes in health care, health informatics is one area where skills are needed to keep up with the demands of the industry.

As the number of programs focusing on health informatics grows, it is important that work-related skills be integrated throughout the curriculum. Despite this growth, a data-driven analysis of US health informatics programs found that only 39% of universities offered health informatics programs and that critical workforce-relevant courses, such as database management and analytics, were absent from many curricula [2]. Undertaking this challenge ensures that academic programs can meet the needs of the workforce by creating a resilient and skills-focused workforce. Health care extends beyond what one learns from textbooks to what students can do when they put those principles into practice. Intentional curricular modifications that develop applied skills and competencies—terms defined more fully in the Skills-First Education and Transferable Competencies section—can prepare a workforce-ready population, reduce talent shortages, and lower the burden of onboarding for employers.

The OECD study notes that labor markets across participating countries are undergoing transformation due to technological innovation, demographic shifts, and an increased focus on job and economic sustainability. These factors are reshaping how skills are recognized and signal the urgent need for programs to align with changes in industry, people, and processes. Job seekers are highlighting their skills to improve their visibility to employers. Although this does not discount the importance of foundational classroom instruction, it does indicate the need for candidates to be work-ready and able to apply acquired knowledge in practice.

Although discussions of competency-based education and workforce alignment exist across health professions education, fewer studies provide operational guidance on how academic programs can translate workforce expectations into curriculum design. This Viewpoint is intended for health informatics program faculty and administrators responsible for curriculum design, program assessment, and workforce alignment. It presents the author’s perspective on how intentional curriculum design can bridge the gap between academic preparation and professional practice in health informatics. This paper aims to contribute to the health informatics education literature in 3 ways. First, it reframes curriculum alignment through a skills-first lens grounded in workforce evidence rather than in abstract competency statements. Second, it positions curriculum mapping as a strategic mechanism for operationalizing workforce alignment across program outcomes, course objectives, and learning activities. Finally, it proposes practical implementation strategies for integrating transferable skills, stackable credentials, and stakeholder engagement into curriculum design.

## Workforce Transformation and the Demand for Applied Skills

Because health care has become increasingly data driven, health care professionals and business leaders must understand the influence of data on health care delivery [2]. Ongoing investment in workforce training related to data knowledge and expertise is integral to expanding workforce skill sets, particularly as AI and machine learning continue to be adopted across clinical and administrative functions [3]. Recent research examining health informatics education has also highlighted the growing importance of AI competencies within academic training. Seba et al [4] demonstrated that integrating generative AI concepts into health informatics curricula can strengthen students’ readiness to engage with emerging digital health technologies and data-driven decision-making environments.

Doll et al [5] emphasize the need for a health data–informed workforce in their article, “A Call for a Health Data-Informed Workforce Among Clinicians,” highlighting the importance of a foundational understanding of data to support scientific inquiry, problem-solving, and decision-making in health care delivery. This knowledge is needed not only at the clinician-patient level but also at the infrastructure and systems level, where sharing health data improves data utility and supports clinical decision-making, thereby facilitating meaningful change.

When considering workforce needs, 2 populations require targeted education and training to remain relevant in the evolving health care ecosystem: existing professionals and emerging professionals. A continuous learning loop is necessary to ensure that the workforce remains aligned with current trends in health care delivery, technology, and research environments. Faculty engagement in continuing education and the intentional transfer of that knowledge to learners play a critical role in ensuring that graduates understand current workforce realities beyond what is reflected in published textbooks.

With the automation of administrative tasks and the integration of technology into health care workflows, skills that complement academic preparation and certification are essential for building a resilient workforce. Bartosik and Wiścicka [6] examined the role of training and certification in graduates’ access to the labor market and found that employers place significant emphasis on certifications, qualifications, courses, and training when hiring new graduates. As labor market expectations continue to evolve, monitoring how these expectations align with educational offerings is essential. This research supports the importance of managing academic programs intentionally and expanding opportunities for graduates to acquire additional competencies and skills as they progress, ultimately increasing their value in a competitive labor market.

Empirical analyses of health informatics job postings further illuminate the gap between academic preparation and workforce demand. Elnoshokaty et al [7] analyzed 831 health informatics job advertisements and found that 87% of positions required a college degree in a related field, while 29% preferred or required professional certification. The most frequently demanded skills included analytics and problem-solving, communication, project management, and critical thinking—competencies that are transferable across industries and closely aligned with the skills-first orientation described in this paper. A parallel analysis of the medical informatics labor market by Schedlbauer et al [8] using web crawling and text mining similarly found a persistent misalignment between competencies taught in academic programs and those demanded by employers, with practical and technical skills consistently underrepresented in curricula. Together, these findings underscore the urgency of using workforce data systematically as a basis for curricular decision-making in health informatics programs.

## Skills-First Education and Transferable Competencies

Skills-first education requires thinking beyond traditional instructional and competency-based frameworks to ensure that learning extends from knowledge acquisition to applied performance. For clarity, competencies are commonly defined in health professions education as the integration of knowledge, skills, and professional attitudes required for effective performance [9]. Skills represent observable abilities that demonstrate applied performance, while knowledge reflects a theoretical understanding of concepts and principles. In this manuscript, a skills-first orientation does not replace competency-based education but rather emphasizes the intentional development and assessment of applied skills within broader competency domains. The intentional integration of competencies ensures that students do not merely understand material conceptually but are also able to execute tasks in real-world contexts. Milana et al [10] discuss the position of adult education advocates who emphasize that skills development is not confined to preparation for a single job, particularly within a continuously shifting labor market. From this perspective, building a sustainable workforce increasingly depends on the ability to demonstrate applied skills and competencies alongside traditional indicators such as degrees, job history, and professional titles.

The emphasis on applied skills in professional education is supported by several strands of educational research. Competency-based education literature emphasizes observable performance as evidence that knowledge and professional attitudes have been successfully integrated into practice [9]. More recent scholarship in health informatics education similarly highlights the importance of developing applied capabilities that enable learners to work with emerging technologies, data-intensive environments, and evolving digital health systems [4]. In this context, the “skills-first” framing used in this manuscript should be interpreted not as a replacement for competency-based education but rather as a practical lens for emphasizing the demonstration of applied capabilities alongside foundational knowledge and professional judgment.

This skills-based orientation is particularly relevant to health informatics education, where learners from diverse academic and professional backgrounds can apply informatics competencies across multiple sectors. Health informatics graduates are not limited to employment in hospitals, technology firms, or research institutions; rather, their skills can be applied wherever data-driven decision-making, system optimization, and information management are needed. This flexibility reflects the transferability of informatics skills across industries and organizational contexts. Table 1 provides examples of transferable skills commonly developed within health informatics education and illustrates how these capabilities can be applied both within health care environments and across other data-driven industries.

**Table 1.** Transferable health informatics skills across sectors.

Skills	Health care application	Cross-industry application
Data analytics	Clinical quality dashboards	Supply chain forecasting
AI evaluation	Clinical decision support tool evaluation	Fraud detection systems
Interoperability	Electronic health record integration	Enterprise system integration
Data governance	Health Insurance Portability and Accountability Act compliance programs	Corporate data governance
Systems optimization	Clinical workflow redesign	Operations management

The development of professional expertise also reflects progressive skills acquisition through experience. The Dreyfus model of skill acquisition [11] describes how learners move from novice to expert through repeated exposure to real-world contexts and decision-making environments. Within this framework, a skills-first orientation does not negate the importance of experiential learning but instead ensures that foundational applied skills are intentionally cultivated during formal education to support this progression.

Supporting this approach requires faculty preparation in assessment development as well as more advanced instructional design and testing strategies. Skills-first education extends beyond field-specific vocational training and instead emphasizes the application of informatics competencies across business, health care, technology, and even hospitality settings. Demonstrating how these competencies transfer across disciplines requires instructional strategies that move beyond traditional curricular boundaries and role-specific examples.

Course- and program-level outcomes designed to assess skill acquisition can be embedded intentionally within instruction and evaluated across multiple learning contexts. For example, foundational skills such as data analysis and interpretation are applicable across industries, while machine learning concepts extend beyond health care into finance, logistics, and operations [12]. Recognizing and assessing these transferable skills expands employment opportunities for graduates and reinforces the broad applicability of foundational competencies developed within health informatics programs [13].

Tushar and Sooraksa [13] examined global employability skills in the 21st-century workplace, identifying recurring skill domains that remain relevant across time and labor market shifts. Through an extensive review, they identified problem-solving, communication, teamwork, adaptability, and a willingness to learn as the most consistently reported skills demanded by employers. The authors emphasize that adapting to a dynamic employment landscape requires individuals who demonstrate not only technical and professional competence but also the ability to engage with emerging technologies, exhibit self-motivation, and remain actively engaged in their work.

Drawing on workforce survey data summarized in their analysis, Tushar and Sooraksa [13] report that a substantial proportion of employers globally experience difficulty recruiting individuals with the skills required for available roles, highlighting persistent skill gaps across industries. This pattern is consistent with findings from health informatics-specific job postings, which similarly point to a shortage of candidates with the analytics and problem-solving skills employers most frequently seek [3]. In response, employers have increasingly adopted strategies such as hiring individuals who already possess relevant skills, investing in the reskilling and upskilling of existing employees, and leveraging automation technologies to meet evolving work demands. These approaches reflect a broader expectation that higher education institutions play a central role in preparing graduates with the competencies needed for today’s labor market.

Despite these expectations, empirical work synthesized by Tushar and Sooraksa [13] continues to demonstrate a misalignment between academic program offerings and labor market requirements, with many curricula struggling to keep pace with rapidly changing workforce needs. A parallel pattern has been documented specifically within medical and health informatics education, where labor market analyses reveal that practical and technical skills remain consistently underrepresented in curricula relative to employer demand [8]. These findings underscore the urgency of aligning program-level outcomes, course objectives, and learning activities in health informatics programs with industry-relevant skills and subject matter that support workforce readiness. These considerations highlight the need for intentional curriculum-level alignment.

## Curriculum Mapping as a Strategic Alignment Tool

It is therefore imperative that academic institutions adopt conceptual and strategic approaches to curriculum mapping that support ongoing alignment with workforce needs and evolving digital health competencies [12,14]. Such approaches can help maintain curricular relevance without constant redesign, reduce onboarding burdens for employers, and better position graduates for successful transition into the workforce. Curriculum mapping provides a structured mechanism for aligning workforce expectations with academic program design. Through this process, program-level outcomes, course objectives, learning activities, and assessment strategies are intentionally aligned with competencies and applied skills identified through workforce analysis. Research examining the alignment of accreditation-defined competencies with industry job postings has demonstrated that gaps persist between what programs teach and what employers require, reinforcing the need for curriculum mapping as an active, ongoing process [15]. Curriculum mapping can thus function as a dynamic planning tool that helps educators identify gaps, redundancies, and opportunities to integrate emerging skills.

Table 2 illustrates how workforce-identified skills can be translated into educational competencies and aligned with program outcomes, learning activities, and assessment strategies. This example demonstrates how workforce-informed curriculum mapping can operationalize emerging skill expectations within health informatics education.

**Table 2.** Example of skills-based curriculum mapping.

Workforce skills	Competency	Industry framework source	Program outcome	Course activity	Assessment
Data analytics	Analyze health care datasets to identify patterns and generate insights that support clinical or operational decision-making	Healthcare Information and Management Systems Society Digital Health Workforce Framework	Analyze health care datasets to support decision-making	Population health data analysis laboratory	Applied analytics project
AI literacy	Evaluate AI-generated outputs used in health care environments	AI health literacy frameworks	Evaluate outputs generated by clinical AI tools	AI case study simulation	Critical evaluation report
Data governance	Evaluate data stewardship practices to ensure regulatory compliance and data quality	American Health Information Management Association data governance framework	Assess data stewardship and compliance practices	Data governance policy review	Policy analysis assignment
Interoperability	Explain and apply interoperability standards used to exchange health care information	Office of the National Coordinator for Health Information Technology interoperability standards	Explain the role of interoperability in health care systems	Workflow mapping using Health Level 7 International and Fast Healthcare Interoperability Resources standards	System integration exercise

## Credential Integration and Stackable Pathways

Adding credential checkpoints throughout an academic program can add value to curriculum design while equipping students with industry-recognized microcredentials and full professional credentials that support employability. These credentials, often grounded in competency-based learning models and issued by higher education institutions or industry partners, provide evidence of learning and verification of specific, acquired skills.

Ahsan et al [14] examined the integration of microcredentials within higher education and highlighted their potential to reshape how learners acquire qualifications aligned with workforce needs. This emerging and disruptive learning model has been positioned as a mechanism for increasing workforce relevance by enabling learners to demonstrate both technical competencies and transferable skills. In addition to supporting hard skills related to technical proficiency, microcredentials may also strengthen day-to-day interactions with people, processes, and systems in professional environments [14]. By aligning educational outcomes with workforce expectations, microcredentials help prepare learners with skills that enhance employability in a rapidly changing labor market.

At the course level, microcredentials serve as a targeted mechanism to foster specific graduate capabilities and make learner competencies visible to employers. This approach supports skills-first education by allowing learners to demonstrate mastery of discrete competencies that are responsive to evolving employment demands.

Microcredentials can also provide added value by supporting preparation for full credentials that signal competence upon program completion. Spohn et al [16] examined credentialing outcomes related to certification pass rates and reported that a portion of candidates who successfully earned full credentials had completed at least 1 additional credential prior to certification. These findings suggest that stacking microcredentials alongside formal academic preparation may reinforce knowledge acquisition and skill development, ultimately supporting success in credential attainment.

Stackable credential pathways allow learners to demonstrate competence across smaller, targeted skill sets while progressing toward full credentials that signal readiness for professional roles. In health informatics and adjacent fields, where applied skills are increasingly required, this layered credentialing approach can strengthen workforce preparation and enhance graduate employability [16].

## Implementation Challenges and Stakeholder Engagement

Integrating health informatics and digital health content into academic curricula presents ongoing challenges, particularly when course development follows linear or static models. Learner-centered implementation approaches instead emphasize cyclical curriculum design, in which development, delivery, and evaluation occur as an iterative process. Regular engagement with stakeholders throughout this cycle supports progressive and innovative health professions education as informatics tools, technologies, and workflows continue to evolve. Structured curriculum development models such as the Kern 6-step approach [17] emphasize systematic needs assessment, goal setting, implementation, and evaluation processes that align closely with the cyclical, iterative approach to curriculum congruency described throughout this paper.

Research examining the integration of health informatics into educational programs has identified several interrelated considerations that influence successful implementation, including curriculum design, development processes, instructional strategies, and ongoing evaluation [12]. Although competency frameworks provide guidance on the knowledge and skills that should be incorporated into health informatics education, academic programs across global contexts continue to experience difficulty translating these frameworks into practice.

Common barriers to implementation include limited executive leadership support, insufficient institutional resources, a lack of faculty with informatics expertise, and broader workforce shortages of trained health informatics professionals [12]. These constraints underscore the importance of involving external stakeholders and industry professionals in curriculum design to support feasibility, relevance, and long-term sustainability. From a practical standpoint, these barriers can be addressed through several logistical strategies. Institutional friction related to curriculum approval processes can be mitigated by engaging faculty governance early and by framing skills-first alignment as an extension of existing accreditation requirements rather than as a wholesale redesign. For example, programs seeking Healthcare Information and Management Systems Society (HIMSS) Academic Education Program (AEP) approval already engage in competency mapping as part of the program approval process, providing a natural foundation for skills-first alignment. These programs map to industry-recognized certifications such as the Certified Associate in Health Informatics and Information Management (CAHIIMS) and the Certified Professional in Health Informatics and Information Management (CPHIMS), offering a direct bridge between academic outcomes and workforce-recognized credentials. Financial costs associated with developing new assessments, acquiring industry credentialing partnerships, or purchasing simulation tools can be managed incrementally by piloting changes within a single course before scaling them program-wide. Programs with limited budgets may also leverage publicly available competency frameworks from organizations such as HIMSS, American Health Information Management Association (AHIMA), and Office of the National Coordinator for Health Information Technology (ONC) as no-cost anchors for curriculum mapping. Faculty development represents a further logistical consideration: not all instructors will have current industry experience or familiarity with skills-based assessment design. Targeted professional development, mentorship from industry-engaged colleagues, and partnerships with professional organizations can support faculty in building this capacity over time. Where dedicated informatics faculty are scarce, adjunct practitioners or industry guest lecturers can supplement instruction in applied skill domains, while full-time faculty focus on foundational and theoretical content.

## Continuous Curriculum Congruency and Program Sustainability

Continuous curriculum congruency checks and program-level evaluation are therefore essential for maintaining the validity and relevance of health informatics education. While some competencies remain foundational and undergo minimal change in underlying theory, health informatics is characterized by frequent shifts in workflows, technologies, and system processes that demand curricular agility. These changes often occur while students are actively enrolled in academic programs, creating challenges for curricula that are reviewed infrequently or remain static over time.

The sustainability and responsiveness of academic programs ultimately determine their ability to remain relevant in a profession that continues to evolve at a rapid pace. Programs that regularly reassess curricular alignment and adapt content to reflect emerging informatics practices are better positioned to prepare graduates for dynamic workforce environments and ongoing transformation.

Practical implementation of a skills-first curriculum alignment model involves several interconnected actions for educators and program leaders. Programs should begin by conducting periodic workforce skill gap analyses using industry reports and employer feedback to identify transferable skill domains relevant to digital health and informatics practice. These findings should then inform the mapping of workforce-relevant skills to program outcomes, course objectives, and learning activities. Applied assessments that allow students to demonstrate competency in real-world scenarios should be embedded throughout the curriculum, alongside the integration of stackable credentials and microcredentials that signal specific skill acquisition to employers. External stakeholders and industry partners should be engaged regularly to validate curriculum relevance, and ongoing curriculum congruency reviews should be conducted to ensure sustained alignment with emerging digital health trends.

## Limitations

This paper offers a conceptual framework and should be interpreted in light of several limitations. The curriculum mapping approach described here is drawn from a synthesis of existing literature rather than from an empirical evaluation of an implemented program, and its effectiveness has not been formally tested across institutional contexts. Institutional variability in resources, faculty expertise, and administrative support may affect the feasibility of full implementation, particularly for smaller or underresourced programs. Additionally, while industry-recognized competency frameworks, such as those from HIMSS, AHIMA, and ONC, provide useful anchors for curriculum alignment, these frameworks evolve over time and vary across regional and organizational contexts. Ongoing validation of credential relevance and employer expectations is therefore necessary to sustain alignment. Future research examining outcomes among graduates from skills-first, credential-integrated programs would strengthen the evidence base for this approach.

## Conclusions: Sustaining Skills-First Health Informatics Education

In a rapidly evolving health informatics profession, skills play a central role in workforce readiness and sustainability. Transferable skills that can be applied across diverse settings and are intentionally integrated into health informatics programs strengthen employability and support the effective use of broad skill sets. Aligning curriculum with workforce-relevant skills, alongside the integration of microcredentials and full credentials, provides a balanced approach to preparing a resilient and adaptable workforce.

This work begins with skilled educators and professionals who are committed to developing and maintaining curricula that remain relevant, timely, and responsive to workforce demands. Curriculum mapping serves as a strategic mechanism to support this alignment, ensuring that graduates are equipped to apply their knowledge and competencies as health informatics professionals in dynamic practice environments. Skills-first education represents an evolution in how learning is designed and delivered rather than a departure from the foundational principles of the profession. As a scalable and adaptable approach, it positions health informatics education to remain agile and effective as the field continues to advance. This perspective highlights how workforce-informed curriculum mapping can serve as a practical mechanism for translating industry skill expectations into sustainable health informatics education design.

## Abbreviations

AEP: Academic Education Program
AHIMA: American Health Information Management Association
CAHIIMS: Certified Associate in Health Informatics and Information Management
CPHIMS: Certified Professional in Health Informatics and Information Management
HIMSS: Healthcare Information and Management Systems Society
OECD: Organisation for Economic Co-operation and Development
ONC: Office of the National Coordinator for Health Information Technology
